# Building community capacity in diabetes care: Perspectives of community health workers

**DOI:** 10.4102/hsag.v30i0.3077

**Published:** 2025-09-16

**Authors:** Tanya Heyns, Mukhethwa A. Muvhungu, Sehlale Mathete, Celia J. Filmalter, Patrick Ngassa Piotie

**Affiliations:** 1 Department of Nursing Science, Faculty of Health Sciences, University of Pretoria, Pretoria, South Africa; 2 School of Health Systems and Public Health, Faculty of Health Sciences, University of Pretoria, Pretoria, South Africa; 3 Diabetes Research Centre, Faculty of Health Sciences, University of Pretoria, Pretoria, South Africa

**Keywords:** diabetes, community healthcare workers, primary health care, capacity building, people living with diabetes, education, emotional support

## Abstract

**Background:**

Diabetes is a global public health concern. Approximately, 79% of people living with diabetes reside in low- and middle-income countries. Community healthcare workers (CHWs) provide basic care to communities, including people living with diabetes in South Africa; however, their contribution still needs to be explored.

**Aim:**

To explore the perceptions of CHWs regarding their roles in diabetes care.

**Setting:**

This study was conducted at primary healthcare clinics in the Tshwane District, located in the northern part of Gauteng province in South Africa, where CHWs form part of ward-based outreach teams.

**Methods:**

Using a descriptive qualitative research design, four focus group interviews were conducted with 32 CHWs with 5 or more years of experience and involved in providing care to people living with diabetes. The Dynamic reading, Engaged code book development, Participatory coding, Inclusive reviewing and summarizing of categories, Collaborative analysis, Translating (DEPICT) approach for collaborative qualitative data analysis was used.

**Results:**

The participants indicated that their roles focused on health education, specifically related to diet, lifestyle changes, medication, involving family and self-care, support and monitoring.

**Conclusion:**

CHWs play an important role in the screening, continuous monitoring and referral of people living with diabetes. It is important that training and support for CHWs are aligned and informed by evidence and the needs of the communities they serve.

**Contribution:**

The results may assist in the development of appropriate competencies, to inform programs and in-service training topics and regulate the practices of CHWs.

## Introduction

The prevalence of diabetes mellitus (DM) is increasing globally and is a major public health concern. In 2021, there were 529 million (95% uncertainty interval [UI] 500–564) people of all ages worldwide living with diabetes, yielding a global age-standardised prevalence of 6.1% (IDF 2022). Between 1990 and 2021, the global age-standardised incidence of diabetes increased by 90.5% (95% UI 85.8–93.6), from 3.2% (3.0–3.5) to 6.1% (5.8–6.5) (IDF 2022). By 2050, more than 1.31 billion (1.22–1.39) people are projected to have diabetes (Ong-Artborirak et al. 2023). Moreover, approximately 80.6% of people living with diabetes reside in low- and middle-income countries (Sun et al. 2022). In South Africa, diabetes was responsible for 95 676 deaths in 2021 (IDF 2022). While 4.2 million South Africans are affected by diabetes, one out of two people living with diabetes remains undiagnosed. According to the International Diabetes Federation, 7.5 million people in South Africa are predicted to have diabetes by 2045 (IDF 2022).

People living with suboptimal controlled diabetes, defined by an HbA1c of more than 7%, may suffer from adverse effects such as diabetic neuropathy, retinopathy, incurable wounds that can lead to lower extremity amputations, worsening mental status and kidney and heart disease (Alam et al. 2021). Diabetes-related complications are expensive (up to a quarter of healthcare expenditure) to treat and are an economic burden for countries that have to purchase medication to ensure that patients receive appropriate care (Chan et al. 2021).

The Lancet Commission on Diabetes recommends changing the ecosystem (structural changes in policy, social systems or the environment), capacity building and improving the clinical practice environment to prevent and control diabetes and other non-communicable diseases (Chan et al. 2021). Building capacity in diabetes care entails empowering an extensive workforce that can reach medically underserved communities and alleviate social determinants of health. Community health workers (CHWs), as integral parts of the health workforce, can significantly enhance community capacity. The positive effects of CHW involvement are well established and supported by a wealth of evidence demonstrating improved diabetes outcomes (Alam et al. 2021; IDF 2022). Community healthcare worker programmes offer tangible, affordable and sustainable solutions, especially in addressing educational needs, care coordination and follow-up of high-risk patients (IDF 2022). In people living with type 2 diabetes, interventions using CHWs have been shown to improve glucose control, blood pressure, diabetes knowledge, perceived competence in managing diabetes and quality of life (Alam et al. 2021). Moreover, a lack of consideration of patients’ societal and personal contexts, individual values and preferences has compromised the implementation of many diabetes management guidelines, particularly in low- and middle-income countries (Chan et al. 2021). Trained CHWs can help people living with diabetes and their families manage stress and solve problems in their daily lives, enhancing their resilience and self-management (Alam et al. 2021).

In response to the diabetes crisis, the South African National Department of Health has adopted a National Strategic Plan for the Prevention and Control of Non-communicable Diseases, including the 90-60-50 cascade for diabetes and hypertension (Basu 2022). The cascade aims to ensure that 90% of all individuals over 18 years of age are aware of their elevated blood pressure or raised blood glucose; 60% of individuals with elevated blood pressure or blood glucose receive an intervention, and 50% of those undergoing interventions achieve control. Goal 3 of the plan focuses on delivering integrated people-centred health services to prevent and control diabetes and non-communicable diseases through a health system strengthening approach (Basu 2022). Community healthcare worker programmes are critical for building community capacity, as recommended by the Lancet Commission on Diabetes (Chan et al. 2021).

The South African primary healthcare system comprises trained healthcare professionals (professional nurses, midwives and medical doctors) and CHWs who assist in ensuring that communities receive adequate health support free of charge (WHO 2020). Community healthcare workers are integral members of primary healthcare teams and are recruited from the communities in which they serve (Mhlongo & Lutge 2019; Schaaf et al. 2020). Community healthcare workers primarily operate in community settings and serve as vital links between healthcare consumers and providers. Their role is to enhance health among groups that have historically lacked access to proper healthcare (Lewis et al. 2019). Community healthcare workers have earned trust within their communities by sharing similar cultures, ethnicities and languages with their clients. This enables them to serve as bridges between community members and the formal health system (Lewis et al. 2019; Hartzler et al. 2018). In South Africa, CHWs are engaged in health promotion and education for individuals living with diabetes (Egbujie et al. 2018; Mhlongo & Lutge 2019). However, issues such as childhood malnutrition, increasing access to birth control and controlling the spread of HIV and TB are often prioritised (Mhlongo & Lutge 2019).

To enhance community capacity for diabetes and achieve the targets outlined in the National Strategic Plan, we examined CHWs’ perceptions of their role in diabetes care and their contributions to achieving diabetes treatment targets.

## Research methods and design

### Study design

This was a qualitative descriptive study that described and documented CHWs’ (insiders) perceptions of their role in diabetes care (Ellis 2019). The study was conducted after the coronavirus disease 2019 (COVID-19) pandemic. We used the Consolidated Criteria for Reporting Qualitative Research (COREQ) (Tong, Sainsbury & Craig 2007).

### Context

The study was conducted at primary healthcare clinics in the Tshwane District, which is located in the northern part of Gauteng province in South Africa and includes urban, semi-urban and rural communities. ‘Ward-Based Outreach Teams’, comprising three to 10 CHWs, are responsible for caring for communities, which include people living with diabetes. Community healthcare workers have no formal medical education but are trained by the Department of Health, NGO’s to provide support and services, including health promotion, to patients in primary healthcare settings. Community healthcare workers have opportunities to enrol in a 60-day home-based course and a 12-month Health Promotion Officer Certificate course, but both offer limited information on diabetes care. Additionally, CHWs receive informal in-service training on diabetes care from healthcare professionals.

### Selection of participants

Purposive sampling was used to identify primary healthcare clinics and participants. Initially, an online discussion was held with the operating manager of the primary healthcare clinics in the Tshwane District to introduce the study. The operating manager then communicated the aim, rationale and benefits of the study to the facility managers of clinics who met the following criteria: (1) employed full-time CHWs, (2) had worked in CHW teams of five or more in the community and (3) were involved in providing care to people living with diabetes. Subsequently, facility managers invited CHWs who had at least 6 months of experience in diabetes care to participate. Once the CHWs agreed to participate, focus group interviews (FGIs) were arranged at a negotiated space, date and time.

### Data collection

Focus group interviews were chosen as the data collection method as they provide an opportunity for participants to build on each other’s ideas. The conversations during the FGIs emphasise engagement and shared experiences, which generate data based on varied viewpoints, expertise and beliefs.

A moderator with experience in conducting focus groups (CF) facilitated the discussions, ground rules were set to respect and be open to all perspectives, only having one person talking at a time and allowing each participant to speak. One of the co-authors (M.A.M., M.P., U.T., N.V.) acted as a co-moderator at each of the interviews. The co-moderators and participants acted as translators when participants were unable to express themselves in English, as they were able to speak the native tongue, the participants and co-moderators reached consensus on the translations during the FGIs. The moderator guided the group discussions, asking questions according to the interview guide and supported participants as they explored the topic with new insights emerging. The moderator ensured that the interview remained focused on the topic and stayed within the allotted time to prevent any impact on client care. The co-moderator assisted with recording equipment, handled unexpected interruptions and took field notes during the interviews. Permission to record the discussion was obtained from the participants. The initial question posed to participants was ‘In your view, please explain your role in diabetes care?’ This was followed by probing questions to encourage expanded responses when appropriate. Participants were encouraged to freely engage in dialogue. The questions were piloted during the first focus group to establish understanding and clarity, and no changes were made; therefore, the data from the pilot session were included in the analysis.

Four FGIs were conducted at the selected primary healthcare clinics in a private room free of disruptions. Each FGI included seven to 11 participants, which is in line with the five to 12 suggested by Gundumogula and Gundumogula (2020), and lasted approximately 45 to 60 min. Data saturation was obtained following the third FGI, as no new data emerged. An additional focus group was held to confirm data saturation.

### Data analysis

The Dynamic reading, Engaged code book development, Participatory coding, Inclusive reviewing and summarizing of categories, Collaborative analysis, Translating (DEPICT) approach, a form of collaborative qualitative data analysis, was used to analyse the data (Flicker & Nixon 2015). The data analysis steps are summarised in Table 1.

**TABLE 1:** DEPICT model: Summary of the steps and activities during data analysis.

Steps	Activities
Dynamic reading	Each team member received one transcribed interview, read the transcript and made notes on the key ideas and/or concepts.
Engaged code book development	A skilled qualitative researcher coordinated a meeting during which a preliminary code book was developed. As a group, we reached consensus around a preliminary code book.
Participatory coding	A copy of the preliminary code book and all four transcripts were then reviewed by each team member.
Inclusive reviewing and summarising of categories	We worked in pairs to develop category summaries, which included generating a list of quotes to support each category.
Collaborative analysing	Each author reviewed the summaries before a meeting with the coordinator. We discussed our findings and reached consensus on new understandings emerging from the data.
Translating	Collaboratively, we created a plan for knowledge translation and reached consensus on how to share findings in recognised journals.

*Source: Adapted from,* Flicker, S. & Nixon, S.A., 2015, ‘The DEPICT model for participatory qualitative health promotion research analysis piloted in Canada, Zambia and South Africa’, *Health Promotion International* 30(3), 616–624

### Trustworthiness

To enhance the trustworthiness of the findings, we used member checking and group debriefing. Credibility was enhanced by conducting multiple focus groups, encouraging participants to share their perspectives, providing an audit trail of what was done and why, and agreeing on transcriptions, codes and themes (Tracy 2010). Reflecting the voices of the participants enhanced confirmability (Polit & Beck 2020). Direct quotes and thick, rich descriptive data were used to enhance transferability (Morrison-Beedy, Côté-Arsenault & Feinstein 2001).

### Ethical considerations

Ethical approval was obtained from the University of Pretoria Faculty of Health Sciences Research Ethics Committee (755/2022). In addition, an ethics clearance certificate was obtained from the Tshwane Research Committee (GP_202301_047). The participants signed informed consent and were informed of their right to withdraw at any time. Minimal personal data were collected, and participants were provided with numbers to ensure confidentiality.

## Results

Thirty-two CHWs participated in four FGIs that were conducted over 2 weeks from 29 August to 07 September 2023. Most of the participants completed Grade 12 (*n* = 31), while one had completed Grade 10. Two participants had a diploma. All the participants attended in-service training sessions at primary healthcare clinics, of which two attended one session on diabetes care. The mean years of experience of the participants was 6.3 years, with a standard deviation of 1.8.

Three themes, namely, health education, support and monitoring and related subthemes and categories, emerged from the data (Table 2).

**TABLE 2:** Themes, sub-themes and categories.

Themes	Sub-themes	Categories
1. Health education	1.1. Managing diabetes	1.1.1 Diet 1.1.2 Implementing lifestyle changes 1.1.3 Medication 1.1.4 Involving the family 1.1.5 Self-care
2. Support	2.1. Emotional support 2.2. Referral	2.2.1 Primary healthcare clinic 2.2.2 Social development
3. Monitoring	3.1. Blood glucose	3.1.1 Glycaemic control monitoring 3.1.2 Early detection of complications 3.1.3 Medication compliance

### Theme 1: Health education

All the participants were aware and ‘proud’ that health education was an important responsibility associated with diabetes care. The participants indicated that health education regarding managing diabetes, specifically related to diet, lifestyle, medication, involving the family and self-care, is imperative for people living with diabetes.

#### Diet

Participants provided health education about the importance of a healthy diet and focused on ways to:

‘[*E*]at healthy while it is still cheap because many people in the community are poor, which does not provide much room for spending on fresh fish and a large variety of fruit and vegetables.’ (FGI 2, participant 1, female)

Clients are advised to ‘plant their own vegetables and fruits to help improve their diet’ (FGI 2, participant 4, female). In addition, the clients are encouraged to use ‘food banks in the community that provide healthy food parcels’ (FGI 1, participant 4, female).

Participants indicated that there were various interventions to encourage healthy eating habits, as demonstrated by the following quotes:

‘[*U*]se cooking without oil and salt as well as reducing portion sizes for better calorie-controlled meals …’ (FGI 2, participant 1, female)

‘[*Y*]ou see … most we tell them not to put eh ba re keng [*oil*] …yes oil in their food … maybe they [*people living with diabetes*] should boil food …’ (FGI 2, participant 3, female)

‘[*Y*]ou [*people living with diabetes*] have to eat healthy … like your vegetables … drink a lot of water and avoid fizzy drinks …’ (FGI 1, participant 4, female)

‘[*T*]hey [*people living with diabetes*] must eat healthy most of the time … if maybe someone is complaining about money to buy food, we tell them to just plant some [*fruit and vegetables*] outside the yard …’ (FGI 3, participant 4, female)

The participants explained that people living with diabetes struggle to eat healthy because of budget constraints:

‘[*B*]ecause I heard that if you are eating too much white pap [*maize meal*], you are at a high risk of getting diabetic … like me, I [*CHW*] am eating pap [*maize meal*] three times a day … it is cheap …’ (FGI 2, participant 2, female)

At times, it was necessary to adjust health education accordingly:

‘[*A*]nd at the end of the day, when you enter into their [*people living with diabetes*] house, you [*CHWs*] assess that the patient is struggling because most of the patients, once you enter in the yard, you assess the environment … also the household then you realize that this person cannot afford what I will say … let me try to give as a person a solution …’ (FGI 4, participant 3, female)

The participants suggested that people living with diabetes plant fruits and vegetables and use social development:

‘[*H*]ere we have got a problem, most of the patients [*people living with diabetes*] are unemployed, so diet is a very difficult problem because when you tell a patient [*person living with diabetes*] to eat specific food, they will tell you that “I cannot afford this”… so we encourage them to do, to do food [*fruit and vegetables*] gardening in their yard …’ (FGI 4, participant 2, female)

‘[*S*]ocial development where they [*government officials*] give food parcels to those who cannot afford to buy food so that they can eat and drink their medication …’ (FGI 2, participant 6, female)

#### Implementing lifestyle changes

The participants voiced that providing health education on lifestyle was important:

‘[*J*]ust to watch their [people living with diabetes] diet and exercise frequently, drink water and …’ (FGI 3, participant 4, female)

‘[*L*]ive a healthy lifestyle … like drinking water, reduce drinking alcohol and smoking … and taking the medication correctly …’ (FGI 4, participant 5, female)

#### Medication

The participants explained their role regarding medication adherence as follows:

‘[*Y*]ou [*CHW*] have to explain to them [*people living with diabetes*] this [*medication*] and this [*medication*] is the same thing, but you must take this [*medication*] … even the doses, you must look at their doses … is it 10 mg or 5 mg but it’s the same package … you must make sure about it [*medication*] …’ (FGI 1, participant 3, male)

The participants explained that their role is to monitor ‘how the medication is treating them [*people living with diabetes*] …’ (FGI 2, participant 4, female) before referring clients to primary healthcare clinics for follow-up. However, the participants raised concerns about their challenges, such as a ‘lack of knowledge in identifying expected side effects’ (FGI 3, participant 4, female) of the medication:

‘Even though he [*male person living with diabetes*] is taking his medication, his manhood is no more working … so we need more knowledge and maybe if they can teach us that it is controllable and then his manhood can still work …’ (FGI 4, participant 4, female)

‘[*T*]here are some questions I cannot answer when I am with the patient [*person living with diabetes*], and they are asking the questions because my knowledge only gets to a certain level … so some of the questions I cannot answer …’ (FGI 1, participant 6, female)

#### Involving the family

Community healthcare workers play an important role in educating the families of people living with diabetes. Community healthcare workers indicated that they taught family members about:

‘[C]ues to look out for that the patient’s [*person living with diabetes*] blood glucose is high’ (FGI 3, participant 2)

‘.. and that family members should ‘encourage the patient [*person living with diabetes*] to monitor their blood glucose level.’ (FGI 4, participant 2, female)

‘[*M*]aintain a healthy diet.’ (FGI 2, participant 5, female)

Which is also supported by:

‘[*W*]e [*CHWs*] advise them [*people living with diabetes*] to teach their family members to help because some they [*people living with diabetes*] say I cannot inject myself … I am afraid …’ (FGI 2, participant 2, female)

However, not all families were interested in supporting people living with diabetes, as evidenced by the following quote:

‘[*N*]ormally with the clients, the families is not really interested in whether they are taking their medication or not … they [*family members*] do not even know the time frames [*taking medication*] … so it is our [*CHWs*] role to teach the family to support the client so that they are taking their medication and make sure that the diet is right as well …’ (FGI 1, participant 6, female)

#### Self-care

The participants indicated that CHWs focus on self-care in the form of foot and wound care, specifically massaging their clients’ feet and assessing whether they had any sores or cuts on their feet:

‘[*Y*]eah to advise them not to wear tight shoes … you know diabetic patients [*people living with diabetes*] if they get a wound takes time to heal … we [*CHWs*] advise them to wear that is open …’ (FGI 2, participant 2, female)

‘[*W*]e [*CHWs*] have to teach them [*people living with diabetes*] that when they have a “sore,” they must take care of their sores because patients with diabetic do not have to have sores … so they must make sure that if they wash their feet … they must make sure that it [*feet*] is dry and everything …’ (FGI 1, participant 5, female)

‘[*S*]ometimes we [*CHWs*] do foot care, like foot care … then we massage their [*people living with diabetes*] feet … we check their sores … then we talk to them to prevent the sore to be big and tell them when they bath they must make sure their feet are dry everywhere … everything is dry …’ (FGI 4, participant 5, female)

### Theme 2: Support

Support was provided in terms of emotional support and the referral of clients to primary healthcare clinics.

#### Emotional support

Although participants indicated that ‘diabetic people often become stressed’ (FGI 3, participant 3, female) and that ‘male patients tend to be short-tempered’ (FGI 4, participant 5, female), nothing was mentioned specifically. One participant indicated that ‘… I do not know how to emotionally support a male patient who mentioned that he is having erectile dysfunction problems …’ (FGI 1, participant 2, female).

#### Referral

The CHWs also indicated their role in referring persons living with diabetes to a higher level of care. Upon asking the participants what they do when they pick up that a client is not doing well, all the respondents replied in a chorus that they refer clients ‘to the clinic’ (FGI 3). Additionally, CHWs may refer their clients to social development and non-governmental organisations:

‘… [*W*]e [*South Africa*] also have NGOs … like when we go to the community … akere [*which means ‘because’ in Sepedi*] … we check with the families if there is someone who is working or not … stuff like that and we refer them … keng [*which means ‘what’ in Sepedi*] to social development where they give food parcels to those who cannot afford to buy food so that they can eat and drink their medication …’ (FGI 3, participant 2, female)

### Theme 3: Monitoring

Community healthcare workers play an important role in monitoring blood glucose and medication compliance among people living with diabetes, fostering effective management and reducing the risk of complications. Regular screening can provide valuable insights into the progression of the disease and guide treatment adjustments.

#### Glycaemic control monitoring

Monitoring glucose levels is important for people with diabetes for several reasons, as it helps to manage the condition effectively, reduces the risk of complications and improves overall health:

‘[*M*]onitor their sugar … and do they have machines … so that they can check their blood glucose every day and monitor it according …’ (FGI 4, participant 4, female)

#### Early detection of complications

The participants felt that they played an important role in preventing complications through early detection:

‘[*S*]ometimes when they do not have the strips … we tell them to check their urine … you know it is sticky [*urine*] … and sometimes it’s also smells [*urine*] …’ (FGI 4, participant 8, female)

‘Sometimes if you check the sugar with the machine …’ (FGI 4, participant 3, female)

#### Medication adherence

The participants noted that men were not as compliant as women. The participants expressed the following as possible reasons for men living with diabetes being ‘difficult’:

‘[*T*]hey [*men living with diabetes*] do not like taking medication …’ (FGI 2, participant 2, female)

‘[*T*]he one that I am talking about [*man living with diabetes*] was so stubborn … akere [*which means ‘because’ in Sepedi*] males do not like going to the hospitals …’ (FGI 3, participant 1, female)

Community healthcare workers used ‘pill counting’ to assess adherence with medication:

‘[*E*]ven pill counting helps because sometimes they forget to take [*pills*] …’ (FGI 3, participant 5, female)

## Discussion

The CHWs expressed three main roles, namely, health education, support and screening. All participants expressed a strong awareness and pride in the important responsibility of providing health education. They indicated that CHWs play an important role in health education by emphasising aspects such as diet, lifestyle, medication and involving the family. Health education is important for patients living with diabetes, as proper management and care can improve glucose control and limit costly long-term complications (American Diabetes Association [ADA] 2023). Additionally, health education allows people to take actions to prevent or reduce their chances of developing disorders and manage their conditions to prevent complications (Rizvi 2022).

Through health education, the unique needs of marginalised and underserved communities can be addressed to achieve health equality (Braveman 2022). People are empowered to make informed decisions regarding behaviour modifications and to gain an increased understanding of their responsibility for their health (WHO 2020). The participants emphasised that individuals living with diabetes should strive for reasonable control, with adherence to medication, diet and a healthy lifestyle being the cornerstone of achieving glycaemic control (Babagoli et al. 2021).

Community-based care providers working under the supervision of a professional nurse deliver medication to individuals living with diabetes and provide instructions on how to administer the medication. This practice is aimed at alleviating congestion at primary healthcare clinics by implementing home delivery systems and promoting medication adherence (Delobelle et al. 2022). In this study, CHWs used ‘pill counting’ to estimate the anticipated number of pills patients should possess during home visits if they had adhered to their dosing regimen and were compliant. Pill counting is regarded as a more objective method for assessing medication adherence compliance (Shiomi et al. 2021).

Participants noted that some family members were unwilling to be involved. Inadequate involvement and support from family members may be detrimental and result in poor diabetes outcomes (Mphasha, Mothiba & Skaal 2022). Patients living with diabetes require family involvement for improved outcomes, coping, improved health status, prevention of complications and adherence compliance with self-care activities. Community healthcare workers play an important role in providing health education on self-care for chronic illnesses, including diabetes (Alsaleh et al. 2021; Andreae et al. 2018), requiring multidimensional and multifaceted practices that promote health and enhance well-being (Scott et al. 2023).

Self-care techniques, such as foot care, have been shown to reduce the occurrence and progression of diabetes-related complications (Alsaleh et al. 2021; Molalign et al. 2021). The participants highlighted physical self-care related to foot and wound care as important for managing individuals living with diabetes. Engaging in proper foot and wound care helps prevent complications such as the development of diabetic foot ulcers and the progression of existing ulcers because of infections and poor wound healing, which may ultimately lead to limb amputations (Karadağ et al. 2019).

Community healthcare workers guide people living with diabetes to engage in healthy behaviours, promote health literacy, foster social support and provide a listening ear (Ong-Artborirak et al. 2023). In our study, CHWs indicated that they offered emotional support to clients, although they were unable to provide specific details on how this support was provided. Participants also highlighted that they encountered challenges when assisting male clients, particularly when addressing issues related to erectile dysfunction. Erectile dysfunction is a common, underestimated problem in men living with diabetes, with a prevalence ranging from 20% to 80% (Jumani & Patil 2020). Therefore, it is essential to enable CHWs to provide sexual health education and provide referrals.

The referral of people living with diabetes is instrumental in their care and management (Egbujie et al. 2018). The Department of Social Development in South Africa is a government agency tasked with safeguarding, fostering development and enhancing the social welfare of South Africans. Its mission is to alleviate poverty and promote social inclusion by crafting and executing policies that establish an enabling environment and reduce poverty stemming from insufficient income because of disability, unemployment or old age. The department collaborates with non-governmental organisations, some of which provide food parcels to individuals who cannot afford food (Department of Social Development 2023). The sustainability of this practice is, however, in jeopardy because of the current political and economic climate. Participants stated that they play a vital role in connecting community members with essential resources. Community healthcare workers facilitate connections between clients and community-based services by making referrals for assistance with food and transportation (Andreae et al. 2018).

Community healthcare workers mentioned that they occasionally refer clients who are at greater risk of developing diabetes to primary healthcare clinics for diagnosis and management (Babagoli et al. 2021). Regular monitoring offers valuable insights into facilitating informed treatment adjustments, enhancing effective management, addressing the progression of the disease and reducing the risk of complications (Alsaleh et al. 2021). Glycaemic control is vital, as a rise in glucose levels is directly linked to the incidence of diabetic complications (Luo et al. 2017). Individuals with poor glycaemic control are at heightened risk of developing coronary heart disease, diabetic foot wounds and retinopathy. These complications often arise because of delayed or inadequate diagnosis and treatment (Duro et al. 2023). Screening for complications has been emphasised and recommended by diabetes management guidelines, including the Society of Endocrinology, Metabolism and Diabetes of South Africa guidelines and the South African Primary Health Care guidelines. These recommendations aim to improve treatment outcomes by detecting and addressing complications in a timely manner (Ajiboye et al. 2022). Such complications exert a significant negative impact on the quality of life of affected individuals and impose a substantial burden on the economy, as increased costs are associated with managing these complications. Therefore, routine screening is critical because it reduces the risk of developing and worsening diabetes-related complications (Ajiboye et al. 2022). Given that individuals with diabetes are supported by CHWs, it is evident that CHWs bear a significant responsibility for timely detection, intervention and referral before complications arise in primary healthcare.

Our study had several limitations. The use of a focus group may have prevented participants from being completely honest, even though confidentiality was assured; however, data saturation from the four focus groups lessened this limitation. In addition, the mental health and psychological support required by people living with could have been explored more in-depth, but can be mitigated with further studies. Researcher bias was minimised by using collaborative data analysis that included discussions about discrepancies and reaching a consensus on findings. We suggest that the study be replicated in primary healthcare clinics throughout South Africa to establish whether CHWs working in different provinces have similar roles. The experiences of people living with diabetes who are receiving care from CHWs and how their role could be improved should be explored. Furthermore, the outcomes of interventions implemented by CHWs in communities should be investigated, as should whether they improve treatment adherence, among other outcomes. Understanding their role in diabetes care is important because this information can be used to develop appropriate competencies, which should inform programmes and in-service training topics and regulate the practice of CHWs.

## Conclusion

Globally and in South Africa, diabetes remains a major concern. Our findings suggest that CHWs aid in diabetes care in the Tshwane District and reveal a deeper understanding and awareness of the important role they play in diabetes care. Community healthcare workers play an important role in screening for diabetes and empowering people living with diabetes and their families through health education and support. Continuous monitoring for complications and referral to a higher level of care are essential competencies, as these actions could decrease the burden on the health system. Future research should explore the cultural aspects related to lifestyle changes, diet, support and gender-specific challenges. It is important to ensure that the training of CHWs is evidence-informed and aligned with the needs of the community. Policy makers should understand the competencies required by CHWs to effectively perform their daily tasks. Additionally, identifying their needs and training gaps is essential for providing adequate support and ensuring their readiness to address the challenges of diabetes care in South Africa.
